# MCL-1 is modulated in Crohn’s disease fibrosis by miR-29b via IL-6 and IL-8

**DOI:** 10.1007/s00441-017-2576-1

**Published:** 2017-02-11

**Authors:** Anke Nijhuis, Renata Curciarello, Shameer Mehta, Roger Feakins, Cleo L. Bishop, James O. Lindsay, Andrew Silver

**Affiliations:** 1 Centre for Genomics and Child Health and National Centre for Bowel Research, Blizard Institute, Barts and The London School of Medicine and Dentistry, Queen Mary University of London, 4 Newark St, Whitechapel, E1 2AT London, UK; 2 Centre for Immunobiology, Blizard Institute, Barts and The London School of Medicine and Dentistry, Queen Mary University of London, 4 Newark St, Whitechapel, E1 2AT London, UK; 3 Department of Histopathology, The Royal London Hospital, London, UK; 4 Centre for Cell Biology and Cutaneous Research, Blizard Institute, Barts and The London School of Medicine and Dentistry, Queen Mary University of London, London, UK

**Keywords:** Fibrosis, miR-29b, MCL-1, microRNA, Crohn’s disease

## Abstract

The miR-29 family is involved in fibrosis in multiple organs, including the intestine where miR-29b facilitates TGF-β-mediated up-regulation of collagen in mucosal fibroblasts from Crohn’s disease (CD) patients. Myeloid cell leukemia-1 (MCL-1), a member of the B-cell CLL/Lymphoma 2 (BCL-2) apoptosis family, is involved in liver fibrosis and is targeted by miR-29b via its 3’-UTR in cultured cell lines. We investigate the role of MCL-1 and miR-29b in primary intestinal fibroblasts and tissue from stricturing CD patients. Transfection of CD intestinal fibroblasts with pre-miR-29b resulted in a significant increase in the mRNA expression of MCL-1 isoforms [MCL-1Long (L)/Extra Short (ES) and MCL-1Short (S)], although MCL-1S was expressed at significantly lower levels. Western blotting predominantly detected the anti-apoptotic MCL-1L isoform, and immunofluorescence showed that staining was localised in discrete nuclear foci. Transfection with pre-miR-29b or anti-miR-29b resulted in a significant increase or decrease, respectively, in MCL-1L foci. CD fibroblasts treated with IL-6 and IL-8, inflammatory cytokines upstream of MCL-1, increased the total mass of MCL-1L-positive foci. Furthermore, transfection of intestinal fibroblasts with pre-miR-29b resulted in an increase in mRNA and protein levels of IL-6 and IL-8. Finally, immunohistochemistry showed reduced MCL-1 protein expression in fibrotic CD samples compared to non-stricturing controls. Together, our findings suggest that induction of MCL-1 by IL-6/IL-8 may surmount any direct down-regulation by miR-29b via its 3’-UTR. We propose that an anti-fibrotic miR-29b/IL-6 IL-8/MCL-1L axis may influence intestinal fibrosis in CD. In the future, therapeutic modulation of this pathway might contribute to the management of fibrosis in CD.

**Electronic supplementary material:**

The online version of this article (doi:10.1007/s00441-017-2576-1) contains supplementary material, which is available to authorized users.

## Introduction

Crohn’s disease (CD) is characterised by transmural inflammation of the affected bowel, which drives disease progression from an inflammatory to a fibrostenosing (stricturing) phenotype (Rieder et al. 2011; Thia et al. 2010). Intestinal wound healing following acute inflammation-induced damage is a complex sequence of events including inflammatory cell activation of subepithelial fibroblasts. This leads to increased collagen deposition and to a decrease in extracellular matrix (ECM) degradation resulting from an imbalance between tissue-degrading matrix metalloproteinases and their inhibitors (Di Sabatino et al. 2009; Graham et al. 1988; Regan et al. 2000). The production of ECM proteins by activated fibroblasts is critical for intestinal wound healing and the contraction of the wound area (Tomasek et al. 2002). Chronic inflammation disturbs this physiological response causing over-production of ECM molecules. This is normally prevented by activation of apoptosis and subsequent removal of the ECM-producing cells. Thus, the over-production of ECM molecules by activated fibroblasts may be a consequence of resistance to apoptosis. Failure of apoptosis promotes the persistence of activated fibroblasts in tissues once repair has been completed. Fibrotic disorders, including pulmonary fibrosis, are often characterised by an overabundance of fibroblasts and fibroblast resistance to apoptosis (Uhal et al. 1998; Huang et al. 2013), indicating that surmounting apoptosis resistance might be an effective treatment strategy for most chronic fibroproliferative diseases. However, the success of such a strategy requires a complete understanding of the anti-apoptotic pathways.

The microRNA (miRNA) miR-29b is one member of the miR-29 family, which comprises miR-29a, miR-29b-1, miR-29b-2 and miR-29c (Chang et al. 2008; Eyholzer et al. 2010; Mott et al. 2010). The miR-29 family precursors are transcribed in two bi-cistronic clusters: miR-29a/b-1 on chromosome 7 (7q32) and miR-29b-2/c on chromosome 1 (1q32). A single nucleotide outside of the seed sequence distinguishes mature miR-29a and miR-29c, whilst miR-29b-1 and miR-29b-2 have identical mature sequences. However, expression of each family member is probably dependent on context, as differential expression and subcellular localisation for individual members has been demonstrated (Hwang et al. 2007), indicating that their functional roles are unlikely to be the same. To date, the miR-29 family has been studied predominantly in the context of cancer and is known for its tumour-suppressor function (reviewed by Wang et al. 2013). This family has also been implicated in the pathogenesis of fibrosis in various organs: the expression of all three members is reduced in fibrosis of the kidney and liver (Qin et al. 2011; Roderburg et al. 2011; Xiao et al. 2012), and miR-29b is down-regulated following myocardial infarction (van Rooij et al. 2008) in the lungs of patients with idiopathic pulmonary fibrosis (Maurer et al. 2010) and in skin fibroblasts of patients with systemic sclerosis (Pandit et al. 2011).

The role of this miRNA family in CD-related fibrosis has not been extensively studied. However, we recently demonstrated reduced miR-29 expression levels in the mucosa overlying strictured gut in CD patients and have shown that TGF-β-mediated up-regulation of collagen in fibroblasts from CD patients is facilitated by reduction of miR-29b (Nijhuis et al. 2014). In addition, loss of miR-29-mediated immunoregulation in CD dendritic cells is linked to the elevated expression of IL-23 associated with this disease (Brain et al. 2013).

**Fig. 1:**
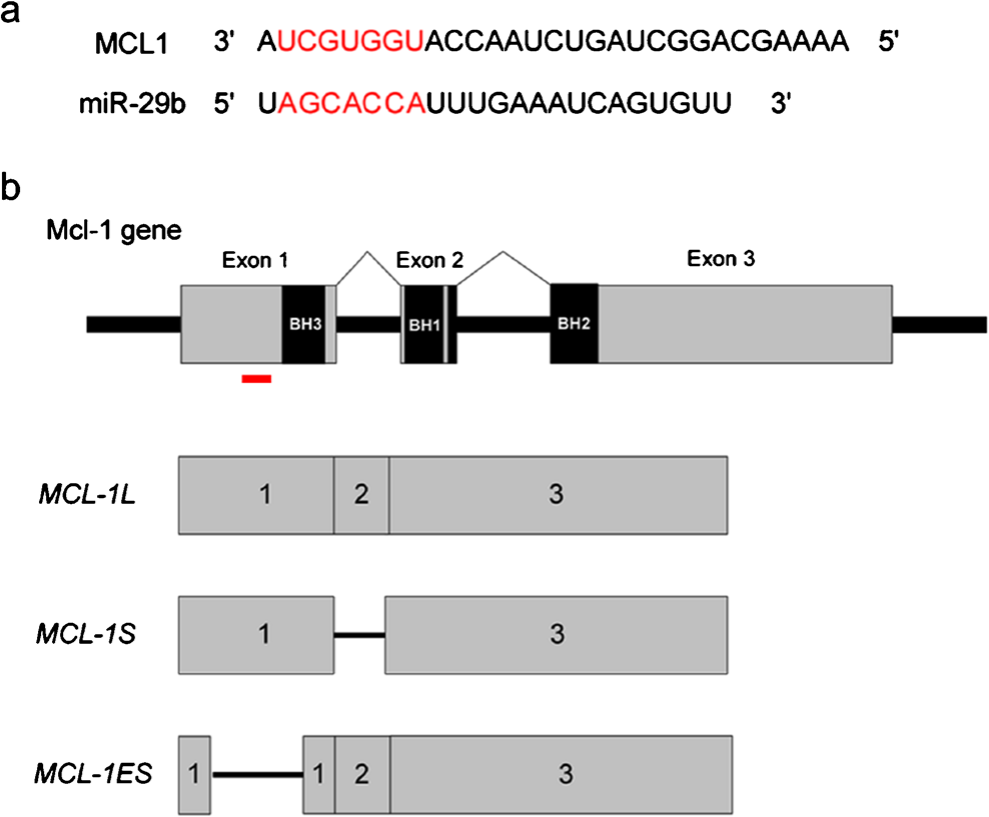
Identification of a single miR-29b binding site with the 3’UTR of *MCL-1*. **a** Predicted binding site of miR-29b within the 3’UTR of *MCL-1* (the 3’UTR is identical for all three isoforms). Nucleotides in *red* indicate complementary binding between the seed sequence of miR-29b and the 3’UTR of *MCL-1*. **b** Schematic overview of MCL-1 gene consisting in three exons. Alternative splicing produces three isoforms: *MCL-1L*, containing the full length of all three exons; *MCL-1S*, exon 2 is lost due to alternative splicing; and *MCL-1ES*, in which the first exon undergoes alternative splicing. The MCL-1L protein containing all three BH domains is part of the anti-apoptotic BCL-2 family, whilst MCL-1S and MCL-1ES have death-inducing properties. *Red line* indicates the epitope of the antibody used to detect MCL-1. The antibody detects both MCL-1L and MCL-1S but not MCL-1ES

A role for miR-29 in resistance to apoptotic cues in CD fibroblasts has not yet been considered. Interestingly, online prediction tools identified *MCL-1*, an anti-apoptotic protein and member of the B-cell CLL/Lymphoma 2 (BCL-2) family, as a miR-29b target in four of the five target prediction sites examined (TargetScan, MiRWalk, miRanda and DIANA Tools; Fig. 1a). Several groups have now validated this prediction demonstrating the binding of miR-29b to the 3’UTR of *MCL-1* through luciferase assays (Garzon et al. 2009; Li et al. 2013; Mott et al. 2007; Roggli et al. 2012; Steele et al. 2010; Xiong et al. 2010). The *MCL-1* gene consists in three exons that undergo alternative splicing to generate three different mRNA transcripts: *MCL-1 long*(L), *MCL-1 short* (S) and *MCL-1 extra short* (ES) (Fig. 1b); MCL-1L is the full-length and most abundant isoform. MCL-1S is expressed at lower levels than MCL-1L (Bae et al. 2000; Bingle et al. 2000; Garzon et al. 2009; Li et al. 2013; Kim 2009; Kim and Bae 2013), whilst MCL-1ES was identified as minimally expressed by RT-PCR (Kim 2009). In cancer, *MCL-1L* was expressed at much higher levels than *MCL-1S* and *MCL-1ES* isoforms; the latter was expressed at low or undetectable levels (Palve et al. 2014). The MCL-1L protein’s anti-apoptotic function is consistent with its 35% homology with the C-terminus of the anti-apoptotic BCL-2 family members and its BCL-2 homology domains (BH)-1, BH-2 and BH-3 (Fig. 1b) (Kozopas et al. 1993). Alternative splicing produces the pro-apoptotic proteins, MCL-1S and MCL-1ES (Bae et al. 2000; Bingle et al. 2000; Kim and Bae 2013). The down-regulation of MCL-1L by miR-29b has been shown to occur predominantly at the protein level (Garzon et al. 2009; Mott et al. 2007; Roggli et al. 2012; Steele et al. 2010; Xiong et al. 2010; Zhang et al. 2011) rather than at the mRNA level (Garzon et al. 2009), indicating that miR-29b might act as a post-transcriptional regulator dependent on disease context and cell type. A pro-apoptotic role for miR-29b in the context of MCL-1 has been shown previously for a number of cellular models and diseases including cancer, diabetes and pre-eclampsia (Li et al. 2013; Mott et al. 2007; Roggli et al. 2012; Xiong et al. 2010; Zhang et al. 2011). However, these investigations did not identify the MCL-1 isoform directly. Deletion of the *Mcl-1* gene in murine hepatocytes resulted in liver cell damage caused by spontaneous induction of apoptosis (Weng et al. 2011). Evaluation of MCL-1 in CD intestinal fibrosis and any inter-action with miR-29b, remains to be investigated.

By modulating expression of miR-29b in intestinal fibroblasts isolated from CD patients, we now show that MCL-1L expression is altered by this miRNA via the cytokines IL-6 and IL-8 and that MCL-1L levels in stricturing CD tissue samples are lower than in non-stricturing CD samples.

## Material and methods

### Isolation of intestinal fibroblasts and culturing

Intestinal fibroblasts were isolated from the mucosa overlying a stricture in resection specimens from individual CD patients and maintained as independent cultures as described previously (Nijhuis et al. 2014). The number of patients from which cultures were isolated is denoted in the figure legends. The studies received the appropriate local Ethics Committee approval (East London REC2) and informed consent was obtained in all cases. Briefly, intestinal mucosa from CD patients undergoing surgery for stricturing disease was used to isolate intestinal fibroblasts. The mucosa was washed twice with HBSS with EDTA (1 mM for 10 min at 37 °C) under gentle agitation to remove epithelial cells. Specimens were cut into smaller pieces and incubated in 20 ml Dulbecco’s modified Eagle’s medium (DMEM) (PAA, UK) with collagenase type 1A (1 mg/ml) and DNase I (10 U/ml) for 45–60 min under gentle agitation at 37 °C in 5% CO_2_ atm. Cells were washed twice with PBS and transferred to a T25 flask and maintained in DMEM supplemented with 10% heat-inactivated FCS, penicillin (100 U/ml) and streptomycin (100 μg/ml) (Pen/Strep). Adherent cells were passaged at 80% confluency at 1:2 to 1:3 ratio using Trypsin-EDTA (PAA). Intestinal fibroblast cultures between passages 4 and 10 were used for functional experiments.

### Transfection of intestinal fibroblasts

Intestinal fibroblasts were seeded overnight in 96-well plates (Nunc, UK) before being transiently transfected with 60 nM negative control siRNA (non-targeting control, NTC #1027281), 60 nM pre-miR-29b, or 120 nM anti-miR-29b (all from Qiagen, UK) using Dharmafect 3 transfection reagent (Dharmacon, USA). Next, 48 h post-transfection, cells were fixed for immunofluorescence. RNA was extracted from 6 wells and combined for qRT-PCR and the culture medium collected for ELISA experiments.

### Stimulation experiments

Intestinal fibroblasts were seeded in 96- or 24-well plates overnight in complete medium. The next day, cells were stimulated with recombinant human 1 or 10 ng/ml IL-6 or IL-8 (R&D Systems, UK) for 4, 8 or 24 h in complete medium. Cells cultured in 96-well plates were then fixed for immunofluorescence and RNA was extracted from cells cultured in 24-well plates.

### RNA extraction and qRT-PCR

Total RNA from intestinal fibroblasts was extracted using the miRNeasy kit (Qiagen) according to the manufacturer’s protocol. RNA concentrations were determined using a NanoDrop Spectrophotometer (NanoDrop Technologies, USA) and 1 μl was run on an agarose gel (1%) to assess RNA quality. RNA samples were reverse transcribed using a High-Capacity-RNA to cDNA kit (Applied Biosystems, USA) in a 20-μl reaction. cDNA was then incubated with TaqMan assays (*MCL-1L/MCL-1ES*, *MCL-1S*, *IL6*, *IL8*, *COL1A2*, *COL3A1* or *GAPDH*) and TaqMan Universal MasterMix (Applied Biosystems) on a 7500 Fast System RealTime PCR cycler (Applied Biosystems) according to the manufacturer’s instructions. The Taqman probe for *MCL-1L* also detects the *MCL-1ES* isoform while there is no commercially available probe for just *MCL-1ES*. A separate probe for selective *MCL-1S* was used. Fold-changes were calculated using the 2^-ΔΔCt^ method normalised to *GAPDH*.

### Immunofluorescence

Intestinal fibroblasts cells were fixed with 3.7% PFA for 15 min at RT before being washed with PBS and permeabilised in 0.1% Triton X-100 (Sigma, UK) in PBS for 20 min. Cells were then washed and blocked for 30 min with 0.25% Bovine Serum Albumin (BSA; Sigma) in PBS before incubation for 2 h with primary antibody MCL-1 (1:250, Cat #32087; Abcam, UK). The antibody used to detect MCL-1 (Cat #ab32087; Abcam) binds epitopes in both the anti-apoptotic MCL-1L and pro-apoptotic MCL-1S isoforms but not MCL-1ES. Cells were washed for 30 min with PBS/BSA (0.25%) and incubated for 2 h with Alexa-Fluor-488 conjugated secondary antibody (1:500; Invitrogen, UK), Hoechst 33342 (1:10,000; Invitrogen) and CellMask Deep Red (1:20,000; Invitrogen) for 2 h. Cells were washed twice with PBS before being imaged on the IN Cell Analyzer 1000 microscope (GE Healthcare, UK) under identical exposure conditions. The IN Cell Developer v.1.8 was used to create a mask overlying the foci. This mask, in combination with Hoechst-positive nuclei, was used to determine the median MCL-1 foci mass within each nuclei [foci mass/nuclei = (total foci pixel intensity x total foci area)/total nuclei count]. Pixel intensities were compared to NTC transfected cells. IN Cell Developer v.1.8 (GE Healthcare) was used to analyse the images.

### Western blotting

Validation of the MCL-1 antibody by western blotting was performed on cell lysates from isolated fibroblasts. The colorectal cancer cell line (CRC) HCT116 was used as a positive control, as MCL-1 has been detected previously in this cell line (Bolesta et al. 2012). Other CRC cell lines used were DLD-1, HT-55, HT-29, SW837 and VACO4S. Lysates were separated using a 4–12% sodium dodecyl sulphate-polyacrylamide gel (Invitrogen). After electrophoresis, proteins were transferred using an electrical field onto PVDF membranes (GE Healthcare). Membranes were blocked for 1 h with 5% non-fat milk in PBS-Tween before being incubated with MCL-1 (1:250) and β-actin (1:50,000; Abcam) primary antibodies overnight at 4 °C in blocking buffer. Goat anti-rabbit or anti-mouse antibodies conjugated to horseradish peroxidase (1:3,000; DAKO, UK) were used as a second layer, before detection using the ECL plus kit (Amersham Biosciences, UK).

### Immunohistochemistry

Formalin-fixed paraffin-embedded 4-μm human tumour sections were dewaxed in xylene and placed in absolute alcohol before application of an endogenous peroxide block for 10 min and rehydrating through graded alcohol concentrations. Antigen retrieval was performed by microwaving sections in a TRIS/EDTA buffer (pH 9.0) for 15 min. Non-reactive staining was blocked using goat serum (1:25 dilution) before MCL-1 primary rabbit antibody application (1:100) for 45 min. Sections were washed in PBS before the secondary goat anti-rabbit antibody (1:250) was applied for 45 min. After further washing, antibody binding was detected using a diaminobenzidine reaction kit (Cat #K3468, DAKO, UK).

### Tissue imaging and scoring

IHC slides were analysed using a light microscope and scored by a pathologist according to stain intensity and proportion of MCL-1-positively staining cells. The percentage of crypt cells and lamina proprial stromal (LPS) cells showing staining at two levels of intensity (1: weak; 2–3: intermediate/strong) was determined. A weighted score from the percentages was then calculated using the following formula: (1 × the percentage staining at intensity 1) + (2 × the percentage staining at intensity 2–3).

### ELISA

Supernatants were taken from intestinal fibroblast cells following transfection with NTC, pre-miR-29b and anti-miR-29b. Cytokines IL-6 and IL-8 were quantified using R&D DuoSet ELISA kits following the manufacturer’s protocol (R&D Systems, USA).

### Statistics

Graphpad Prism analysis software was used to calculate significance using a two-tailed Student’s *t* tests. A *p* value of <0.05 was considered statistically significant.

## Results

### MiR-29 up-regulates the MCL-1L/ES mRNA transcript to a greater extent than MCL-1S

**Fig. 2:**
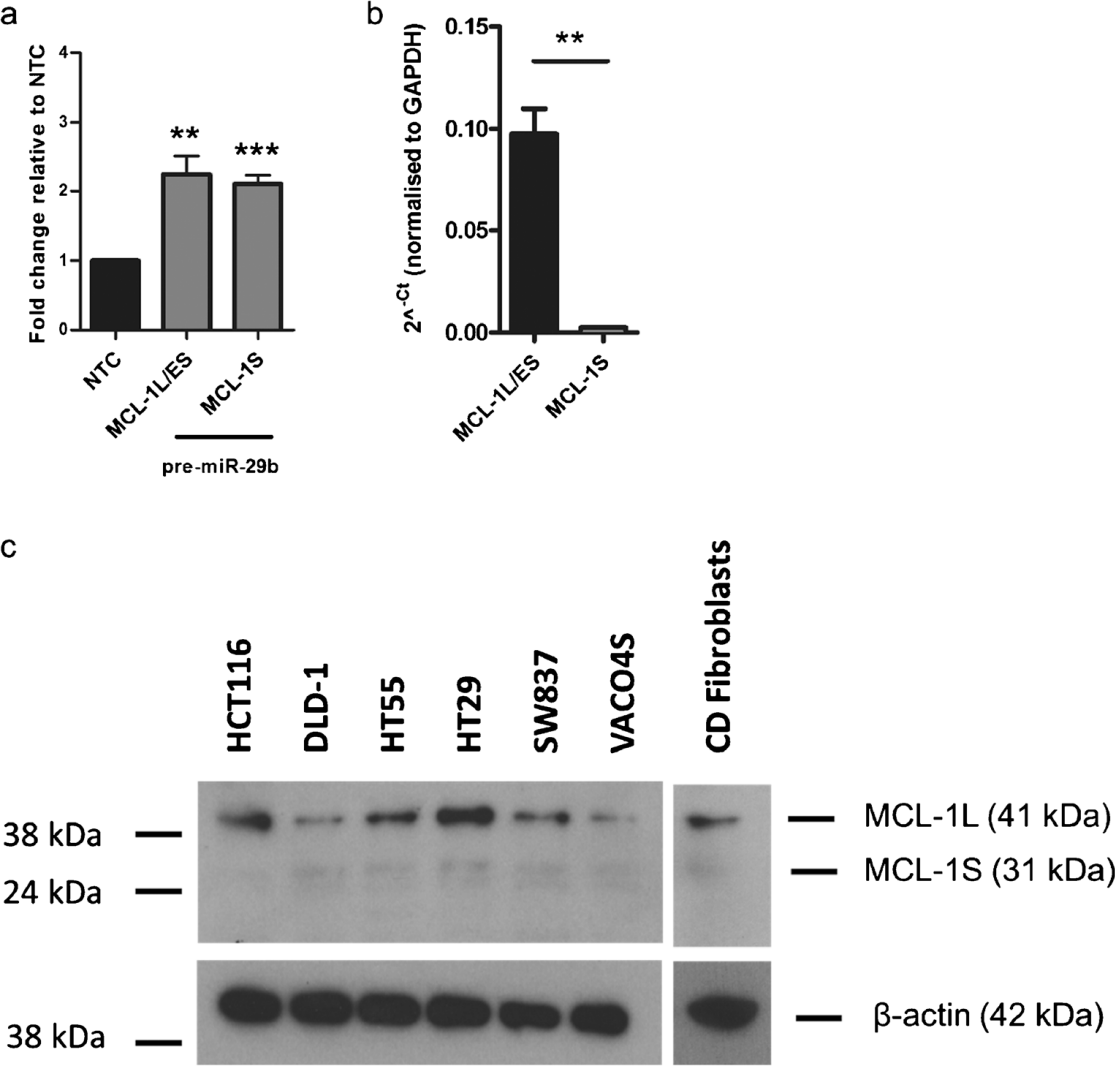
Expression of *MCL-1* mRNA following miR-29b tranfection and MCL1 protein in CD fibroblasts. **a** Intestinal fibroblasts transfected (*n* = 5, each from a different individual) with NTC or pre-miR-29b. Fold change in expression of *MCL-1L/ES* and *MCL-1S* measured by qRT-PCR. **b** Expression values (2^^-Ct^) for *MCL-1L/ES* and *MCL-1S* normalised to *GAPDH. Bars* represent mean values with SEM. **p < 0.01, ***p < 0.001. **c** Cell lysates from six CRC cell lines (HCT116, DLD-1, HT29, HT55, SW837 and VACO4S) and intestinal fibroblasts from CD patients were subjected to western blotting. An antibody against both MCL-1L and MCL-1S and β-actin was used at 1:200 and 1:50,000, respectively. The molecular weights for MCL-1L and MCL-1S are 41 kDa and 31 kDa, respectively and both isoforms are detected in CRC lines and CD fibroblasts. The molecular weight of MCL-1ES is 25 kDa and was not detected. A very faint non-specific band about 30 kDa is also detected but only in the CRC lines not the CD fibroblasts

To assess the relationship between miR-29b and *MCL-1* mRNA expression, intestinal fibroblasts were transfected with NTC or pre-miR-29b and fold change in *MCL-1* mRNA was determined relative to the NTC control. A significant increase in *MCL-1* mRNA transcript levels was observed in intestinal CD fibroblasts (*MCL-1L/ES*, *p* = 0.004; *MCL-1S*, *p* = 0.0008; Fig. 2a). It should be noted that, although both *MCL-1L* and *MCL-1S* transcripts were detected in the isolated fibroblasts, the expression of *MCL-1L* was expressed 43 times higher than *MCL-1S* (*p* = 0.0044; Fig. 2b). The probe for *MCL-1L* also detects *MCL-1ES* but the later is likely expressed at much lower levels than *MCL-1L* and *MCL-1S* as reported previously (Kim 2009; Kim and Bae 2013; Palve et al. 2014). These data show the up-regulation of MCL-1/ES transcripts following transfection of miR-29b in intestinal fibroblasts.

### MCL-1L is the predominant protein isoform in CD intestinal fibroblasts

Next, the expression of the MCL-1 protein in intestinal fibroblasts was investigated. A dominant band at 41 kDa that correlated with the molecular weight of MCL-1L was confirmed (Fig. 2c; Supplementary Table S1). By contrast, the 31-kDa band equivalent to the molecular weight for MCL-1S was much fainter. Image J software used to quantify MCL-1-positive bands showed that the MCL-1L isoform was detected at higher levels than MCL-1S in CD fibroblasts and CRC cell lines (Fig. 2c). Taken together, the mRNA and protein data indicate that the most common isoform present in CD fibroblasts is the anti-apoptotic MCL-1L (Fig. 2).

### MiR-29 up-regulates *MCL-1L* protein

**Fig. 3:**
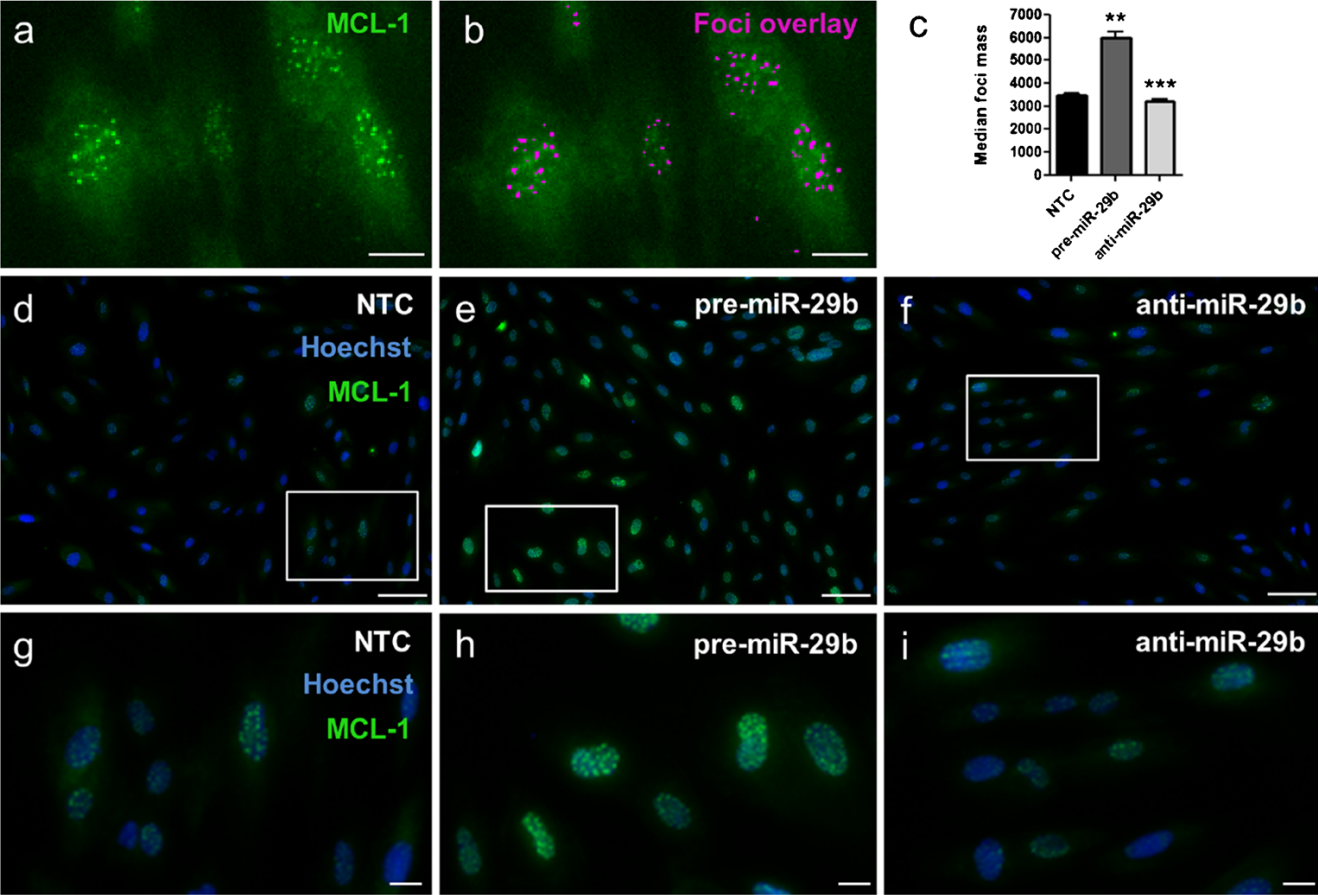
MCL-1L protein expression following miR-29b transfection. Intestinal fibroblasts (*n* = 3, each from a different individual) were transfected with NTC, pre-miR-29b and anti-miR-29b for 72 h. Cells were fixed and stained with Hoechst 33342 (*blue*) and an antibody against MCL-1 (*green*) **a**, **b** Representative immunofluorescence images of CD fibroblasts transfected with NTC to illustrate the generation of the MCL-1L foci overlying mask (**b**). **c** Fold change in median foci mass/nuclei following transfection with pre-miR-29b or anti-miR-29b relative to NTC. **d**–**f** Representative images of MCL-1L foci following transfection with NTC, pre-miR-29b or anti-miR-29. Rectangles outline digital zoomed area. *Bars over columns* mean values±SEM. **p < 0.01, ***p < 0.001. Zoomed images **g**-**i **20 µm bars, original images **d-f **100 µm

To explore further the effects of miR-29b on MCL-1L in intestinal fibroblasts, protein expression and localisation was determined by immunofluorescence using the MCL-1 antibody that detects predominately the MCL-1L isoform in the CD fibroblasts (Fig. 2c). Fibroblasts were transfected with NTC, pre-miR-29b or anti-miR-29b and the MCL-1L protein was found localised in discrete nuclear foci (Fig. 3a, b). Intestinal fibroblasts transfected with pre-miR-29b generated a significant increase in the median MCL-1L-positive foci mass, whilst cells transfected with anti-miR-29b resulted in a significant decrease in median foci mass compared to cells transfected with NTC (pre-miR-29b, *p* = 0.0029; anti-miR-29b, *p* = 0.0003; Fig. 3c). Representative images are shown in Fig. 3d–f. In addition, over-expression of miR-29 increased the number of MCL-1 foci (*p* = 0.0198; Supplementary Fig.S1)

Taken together, these data demonstrated that miR-29b induced an increase of the anti-apoptotic MCL-1L form at both the mRNA and protein level in intestinal fibroblasts. In support of this, transfection with pre-miR-29 or anti-miR-29 did not alter the number of cells compared to NTC transfected cells (pre-miR-29, *p* = 0.773; anti-miR-29b, *p* = 0.784; Supplementary Fig. S2), indicating that the pro-apoptotic forms of MCL-1 (MCL-1S and MCL-1ES) protein are not induced by miR-29b.

### miR-29b up-regulates MCL-1L potentially through IL-6 and IL8

**Fig. 4:**
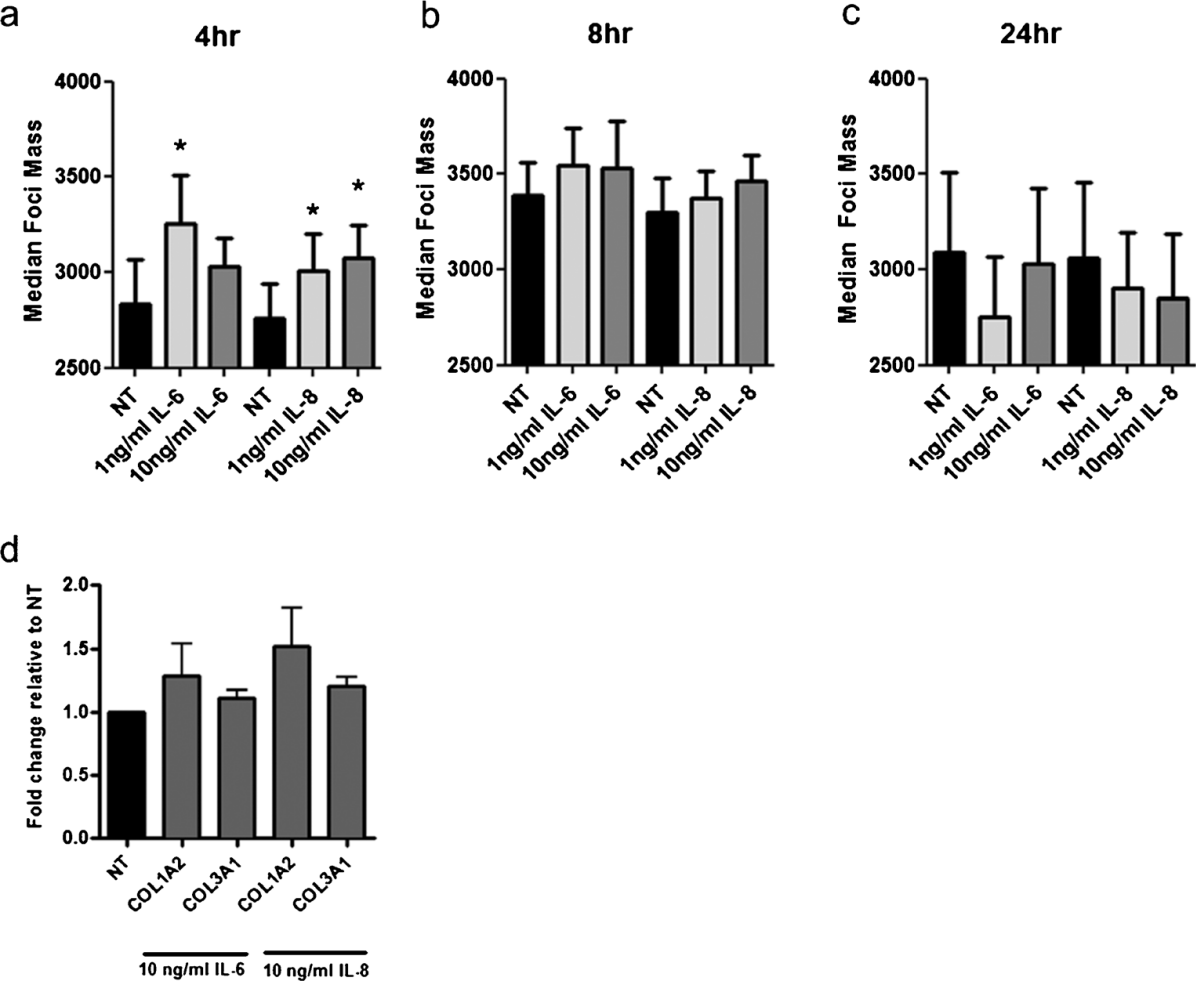
MCL-1L protein expression is induced by IL-6 and IL-8. Intestinal fibroblasts (*n* = 5, each from a different individual) were treated with 1 or 10 ng/ml of IL-6 or IL-8 for 4, 8 and 24 h. Cells were fixed stained with Hoechst 33342 and an antibody against MCL-1 and median foci mass quantitated. **a**–**c** Median MCL-1L mass/nuclei following treatment with 1 or 10 ng/ml IL-6 or IL-8, relative to NT at 4 h (**a**), 8 h (**b**) and 24 h (**c**). **d** Intestinal fibroblasts (*n* = 3, each from a different individual) were treated with 10 ng/ml of IL-6 and IL-8 for 48 h. qRT-PCR was performed on extracted RNA and mRNA levels *COL1A2* and *COL3A1* determined. The graphs represent fold change relative to the NT control. *Bars above columns* mean±SEM, *p < 0.05

We hypothesised that the up-regulation of MCL-1 observed following miR-29b transfection occurs via two known up-stream regulators of MCL-1, interleukin (IL)-6 and IL-8 (Puthier et al. 1999a, b; Sarkar et al. 2012). First, we examined the regulatory effect of IL-6 and IL-8 on MCL-1. Intestinal fibroblasts from CD patients were treated with IL-6 and IL-8 (1 or 10 ng/ml) for 4, 8 or 24 h and MCL-1L foci quantitated. Fibroblasts treated with either IL-6 or IL-8 for 4 h up-regulated the median mass of MCL-1-positive foci (1 ng/ml IL-6, *p* = 0.029; 10 ng/ml IL-6, *p* = 0.509; 1 ng/ml IL-8, *p* = 0.025; 10 ng/ml IL-8, *p* = 0.015; Fig. 4a). Stimulation for longer than 4 h (8 or 24 h; 1 or 10 ng/ml) diminished this up-regulation (all *p* values >0.08; Fig. 4b, c). These data support the hypothesis that Il-6 and IL-8 up-regulate MCL-1L protein expression in CD intestinal fibroblasts.

To identify whether the miR-29b/IL-6/IL-8 axis affects the collagen genes previously shown to be down-regulated in fibroblasts from CD patients by miR-29b (Nijhuis et al. 2014), mRNA expression of both *COL1A2* and *COL3A1* was measured following stimulation with IL-6 or IL-8 (10 ng/ml). Fold change in expression relative to non-treated (NT) fibroblasts demonstrated no change in the expression of either *COL1A2* or *COL3A1* following stimulation with IL-6 (*COL1A2*, *p* = 0.1988; *COL3A1*, *p* = 0.1997; Fig. 4d) or IL-8 (*COL1A2*, *p* = 0.2274; *COL3A1*, *p* = 0.1222; Fig. 4d).

**Fig. 5:**
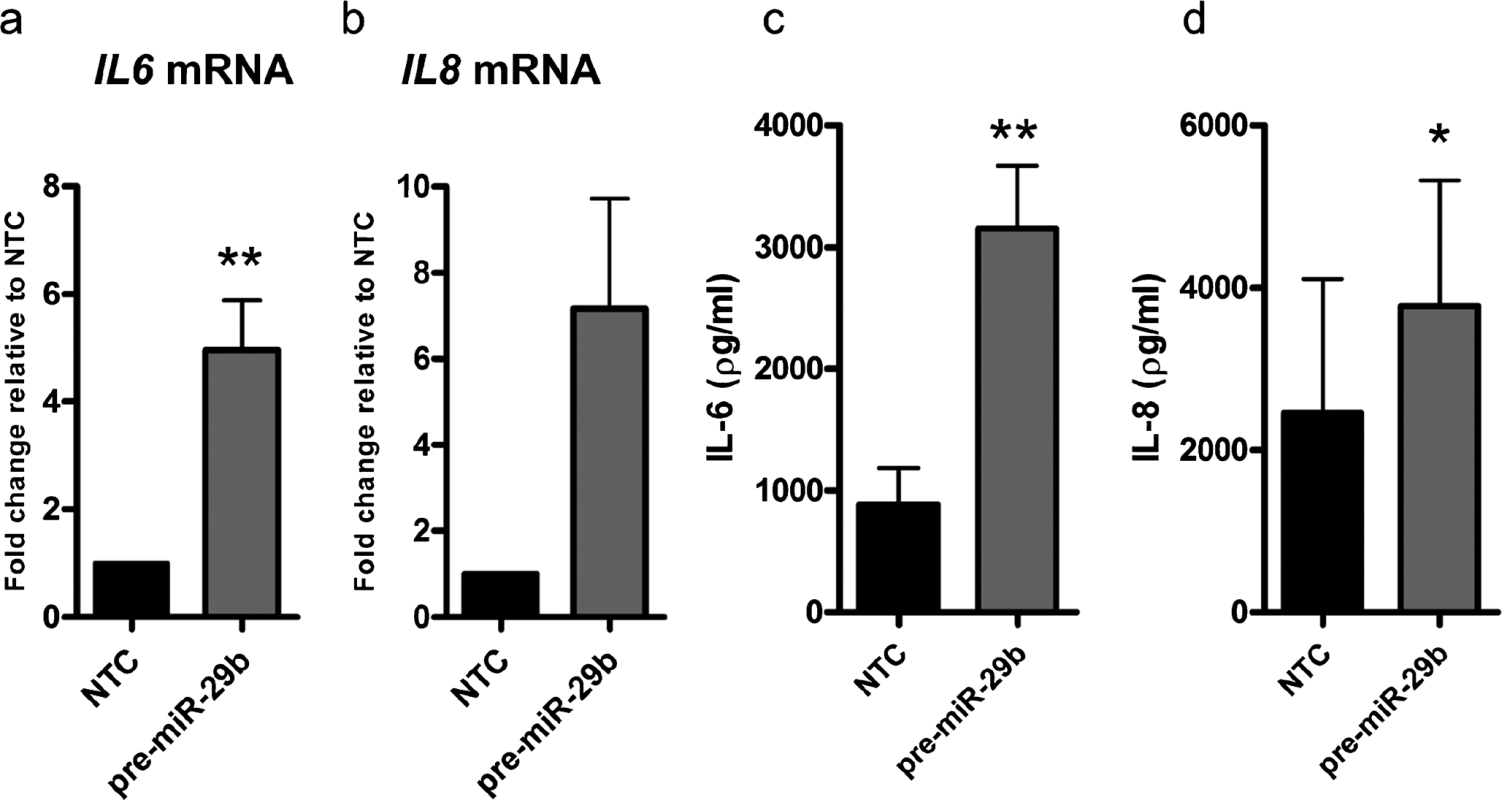
miR-29b up-regulates IL-6 and IL-8. **a, b ** Intestinal fibroblasts (*n* = 6, each from a different individual) were transfected with NTC or pre-miR-29b for 48 h. The graphs represent the fold change in expression of *IL6* (**a**) and *IL8* (**b**) mRNA relative to the NTC control as measured by qRT-PCR. **c**, **d** Supernatant was collected from fibroblasts transfected with NTC or pre-miR-29b after 48 h. The *graphs* represent the production of IL-6 (**c**) and IL-8 (**d**) as measured by ELISA. *Bars above columns* mean values±SEM. *p < 0.05, **p < 0.01

To further test the hypothesis that miR-29b up-regulates MCL-1 via IL-6 or IL-8, intestinal fibroblasts were transfected with NTC and pre-miR-29b. *IL6* and *IL8* mRNA expression was assessed via qRT-PCR and normalised to the housekeeping gene *GAPDH*. Fibroblasts transfected with pre-miR-29b showed a significantly increased fold change of *IL6* compared to NTC transfected cells (*p* = 0.0077; Fig. 5a). *IL8* mRNA levels were also up-regulated by pre-miR-29b and approached significance (*p* = 0.06; Fig. 5b). ELISA was then used to measure IL-6 and IL-8 production in the supernatant of fibroblasts following transfection. Levels of both cytokines were increased significantly by fibroblasts transfected with pre-miR-29b compared to NTC (IL-6, *p* = 0.0027; IL-8, *p* = 0.0268; Fig. 5c, d). These results demonstrate that miR-29b up-regulates the expression of *IL6* and *IL8* at the mRNA level, although this change did not quite reach significance for *IL8* (Fig. 5) and their release into the supernatant.

### MCL-1 expression is reduced in fibrotic CD tissue

**Fig. 6:**
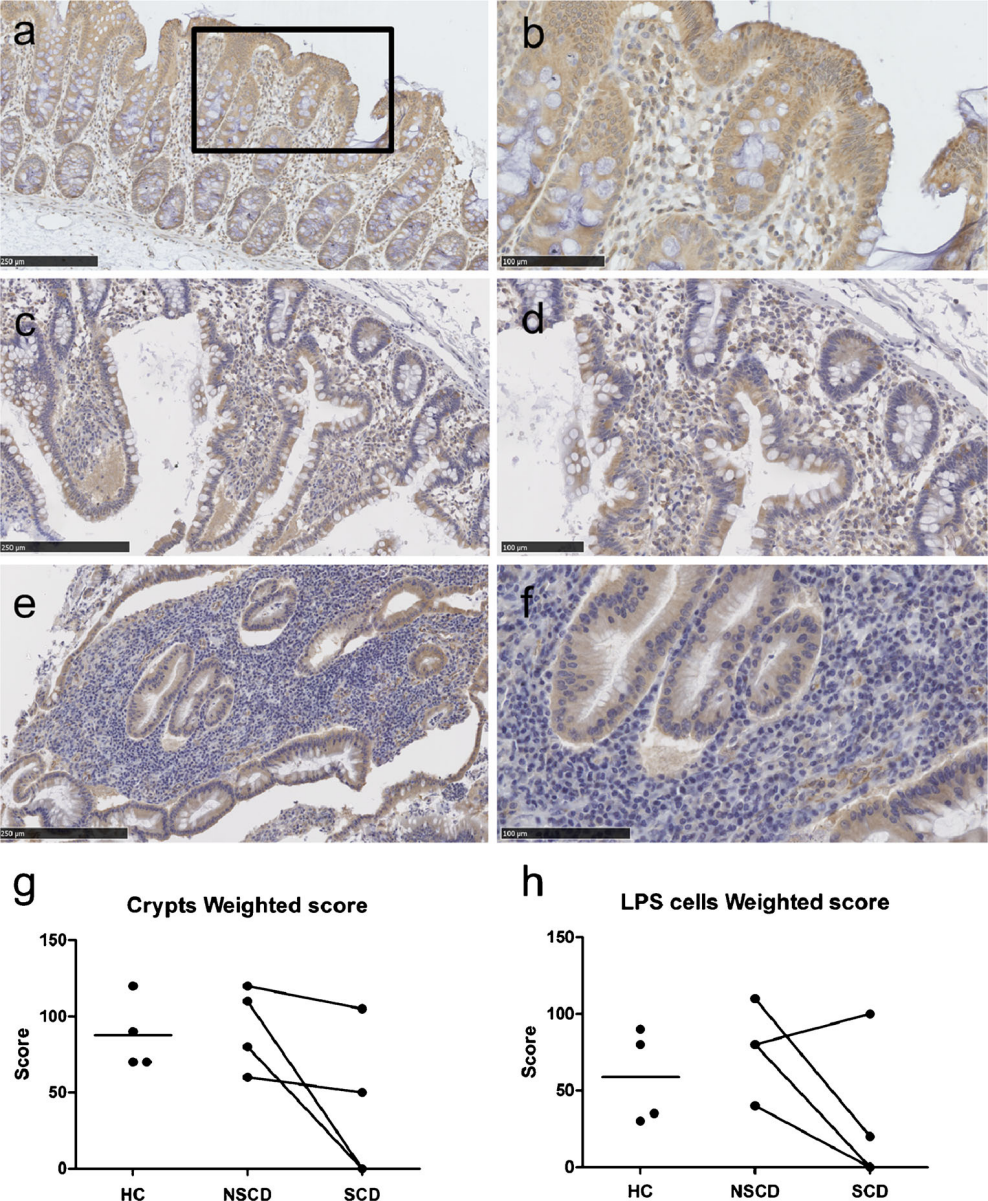
MCL-1 protein expression in CD tissue samples. Immunohistochemical staining for MCL-1 in human ileal tissue: four paired NSCD and SCD tissue samples and four samples from healthy control patients. **a, b** Mucosa from a healthy control patient. Staining in both epithial cells and lamina proprial stromal (LPS) cells. **c**, **d** Mucosa from a patient with non-stricturing CD showing extensive cytoplasmic MCL-1 expression by crypt epithelial and LPS cells. **e**, **f** Mucosa from a patient with stricturing CD showing extensive staining in the epithelial cells but little or no expression by LPS cells. Digitally zoomed areas on the right (**b**, **d**, **f**). **g**, **h** The weighted score from the intensity percentages is shown for both crypt cells (**g**) and LPS (**h**) cells

We have shown previously that miR-29b was down-regulated in stricturing CD (SCD) compared to non-stricturing (NSCD) (Nijhuis et al. 2014). Based on our finding here, we hypothesised that anti-fibrotic MCL-1 expression will also be reduced in SCD intestinal tissue resected from CD patients. Immunohistochemistry was performed on four healthy control samples and four paired SCD and NSCD samples (Fig. 6a–f). A decrease in staining intensity of both crypt and LPS cells in SCD compared to NSCD tissues was found, while the levels of MCL-1 in control gut was similar to NSCD tissues (Fig. 6g, h). The reduction in MCL-1 expression in stricturing CD tissue provides in vivo support for a role for the anti-fibrotic miR-29b/MCL-1 axis in CD.

## Discussion

It has been reported that direct targeting of the 3’UTR of MCL-1L by miR-29 leads to its down-regulation in cell lines (Garzon et al. 2009; Li et al. 2013; Mott et al. 2007; Roggli et al. 2012; Steele et al. 2010; Xiong et al. 2010). In contrast, we found that transfection with pre-miR-29b of primary fibroblasts isolated from CD patients resulted in an increase of MCL-1L at both mRNA and protein levels. In addition, we have shown previously that miR-29b is anti-fibrotic in CD intestinal fibrosis (Nijhuis et al. 2014). This accords well with both our observation here, that this miRNA up-regulates MCL-1L in fibroblasts, and the reported anti-fibrotic properties in the liver (Kahraman et al. 2009; Vick et al. 2009; Weng et al. 2011). Hence, we hypothesised that the up-regulation of MCL-1L via miR-29b in intestinal CD fibroblasts is indirect. Moreover, that the mediator(s) through which this up-regulation is affected is strong enough to overcome/override the modest direct down-regulation that miR-29b may exert on MCL-1L through its 3’UTR.

One of the most potent inducers of MCL-1 is IL-6 (Puthier et al. 1999a, b), a classic pro-survival cytokine that is crucial in mounting an effective immune response. In addition, recent studies have shown that IL-6 expression is up-regulated in renal fibrosis in mice (Fielding et al. 2014) and that this cytokine can induce the expression of collagen I (O’Reilly et al. 2014). Furthermore, IL-6 has been implicated in a variety of fibrotic conditions via alternative trans-signalling pathways (O’Reilly et al. 2012). The up-regulation of MCL-1 by IL-6 is most likely due to the activation of the STAT3 transcript factor (reviewed in Aggarwal et al. 2009). A second cytokine, IL-8, can also increase the expression of MCL-1 (Puthier et al. 1999b) and elevated serum levels of IL-8 are associated with fibrosis in chronic liver disease (Nobili et al. 2004). In this study, we confirmed the up-regulation of MCL-1 by IL-6 and IL-8 in intestinal fibroblasts at the protein but not mRNA level (Fig. 4). Crucially, transfection with pre-miR-29b significantly increased the production of IL-6 and IL-8 (Fig. 5), identifying a functional interplay between miR-29b, IL-6/IL-8 and MCL-1L. Moreover, the down-regulation of MCL-1 by miR-29b can be abrogated by IL-6 (Zhang et al. 2001). This suggests that the induction of MCL-1 by IL-6/IL-8 may surmount its direct down-regulation by miR-29b via 3’-UTR of MCL-1. Overall, our observational data led to a hypothesis that an anti-fibrotic miR-29b/IL-6 IL-8/MCL-1 axis exists in CD intestinal fibrosis.

**Fig. 7:**
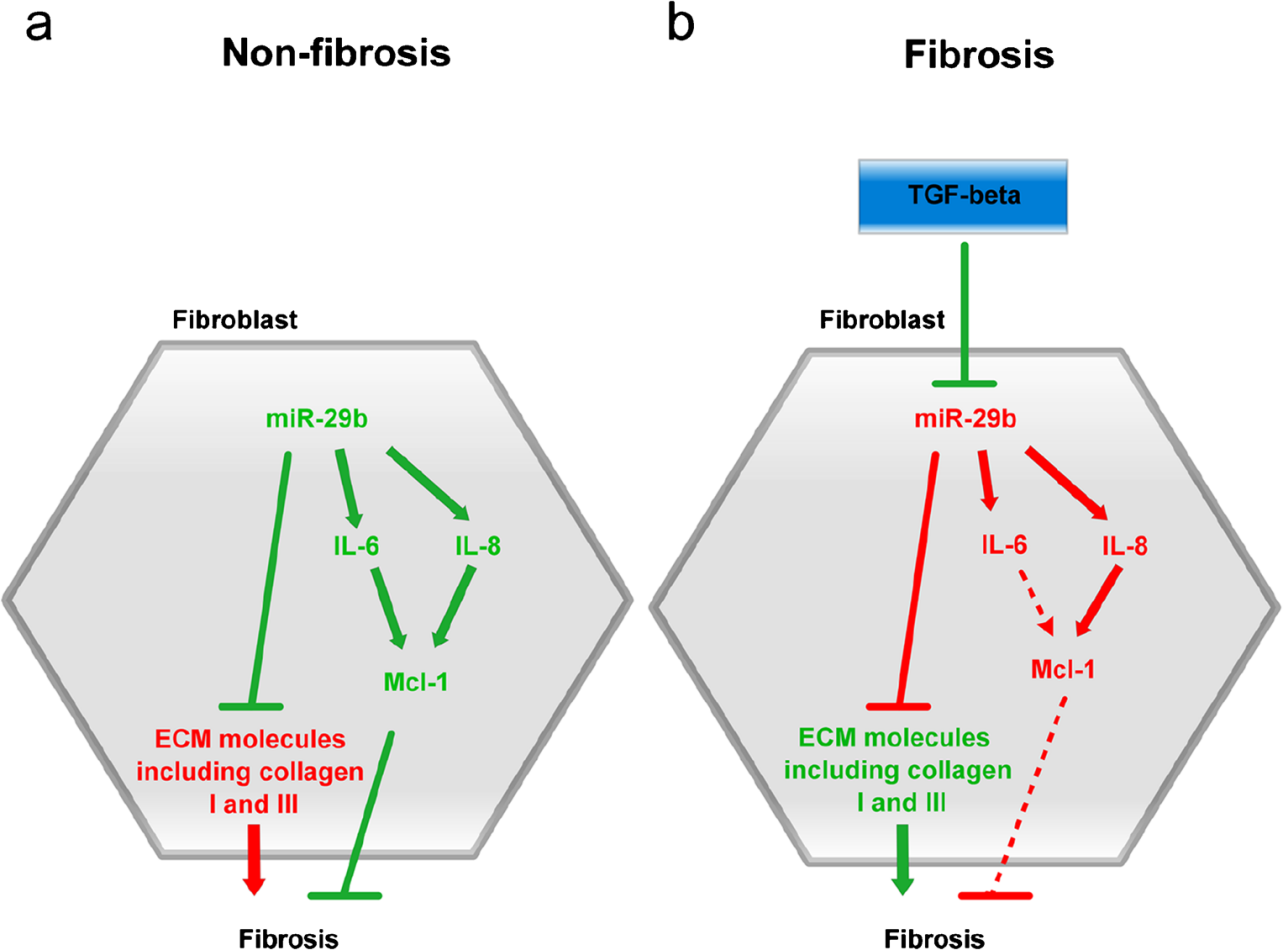
Proposed model of the role of miR-29b in CD fibrosis. TGF-β is a potent pro-inflammatory cytokine. TGF-β modulates fibrosis through down-regulation of miR-29b, resulting in increased deposition of collagen and therefore fibrosis. In CD fibrosis, additional down-stream pathways of miR-29b are as yet unknown. Up-regulation of anti-fibrotic mediator MCL-1 by miR-29b may potentially be mediated through IL-6 and IL-8. Up-regulated genes are in *green*, down-regulated genes in *red*

To our knowledge, this is the first time MCL-1 expression has been investigated in tissue samples from CD patients. In support of our findings, Liu and colleagues showed that MCL-1 is down-regulated in intestinal tissues from patients with ulcerative colitis and mice with dextran sodium sulfate-induced colitis (Liu et al. 2010). The decrease in MCL-1 in fibrotic CD tissue samples supports our previous observations of reduction of miR-29b expression in stricturing CD (Nijhuis et al. 2014). A hypothetical model of how TGF-β may exert its pro-fibrotic action through the miR-29b/IL-6/MCL-1 axis is shown in Fig. 7. We propose a mechanism whereby the up-regulation of the anti-fibrotic mediator MCL-1 by miR-29b is mediated through IL-6 and IL-8. The pro-fibrotic cytokine TGF-β modulates fibrosis through down-regulation of miR-29b, resulting in increased deposition of collagen and therefore fibrosis. Hence, the down-regulation of miR-29b results in reduced MCL-1 expression. Further functional experiments are warranted to confirm this anti-fibrotic pathway in vivo. The latter may well require the development of new animal models including conditional modulation of miR-29b expression in the mouse intestine using a suitable knock-in construction. In the future, therapeutic modulation of this pathway to reduce fibrosis might be possible.

## Electronic supplementary materials

Below is the link to the electronic supplementary material.

Supplementary Figure S1 (GIF 322 kb)

High resolution image (TIF 8448 kb)

Supplementary Figure S2 (GIF 51 kb)

High resolution image (TIF 9377 kb)

Supplementary Table S1 (DOC 28 kb)

## Abbreviations

BCL-2: B-cell CLL/Lymphoma 2
BH: BCL-2 homology domains
CD: Crohn’s disease
DMEM: Dulbecco’s modified Eagle’s medium
LPS: Lamina proprial stromal
MCL-1: Myeloid cell leukemia-1
MCL-1L: Myeloid cell leukemia-1 long isoform
MCL-1S: Myeloid cell leukemia-1 short isoform
MCL-1ES: Myeloid cell leukemia-1 extra short
miRNA: MicroRNA
NSCD: Non-stricturing Crohn’s disease.
NT: Non-treated
NTC: Non-targeting control
SCD: Stricturing Crohn’s disease
