# Supplementary figures and images for: MCL-1 is modulated in Crohn’s disease fibrosis by miR-29b via IL-6 and IL-8
Source: Cell Tissue Res. 2017 Feb 11;368(2):325–35. doi: 10.1007/s00441-017-2576-1 (PMC5397660; doi:10.1007/s00441-017-2576-1)

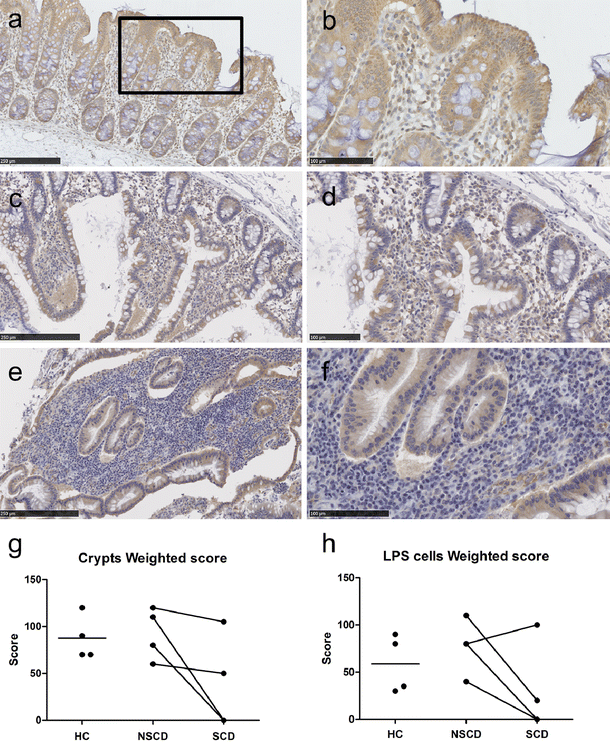

Supplement: Supplementary file 1 — (GIF 322 kb) [file 441_2017_2576_Fig8_ESM.gif]

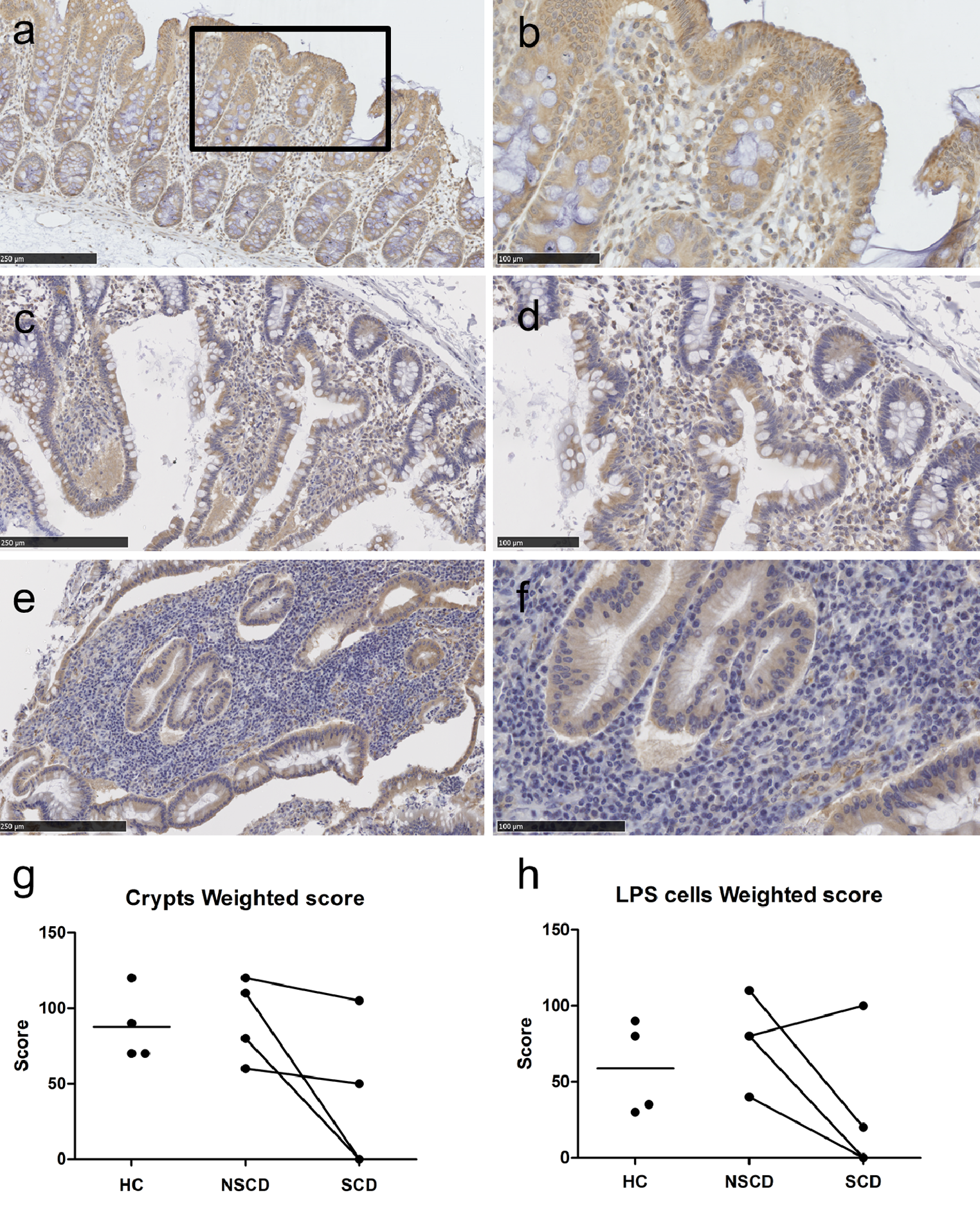

Supplement: Supplementary file 2 — High resolution image (TIF 8448 kb) [file 441_2017_2576_MOESM1_ESM.tif]

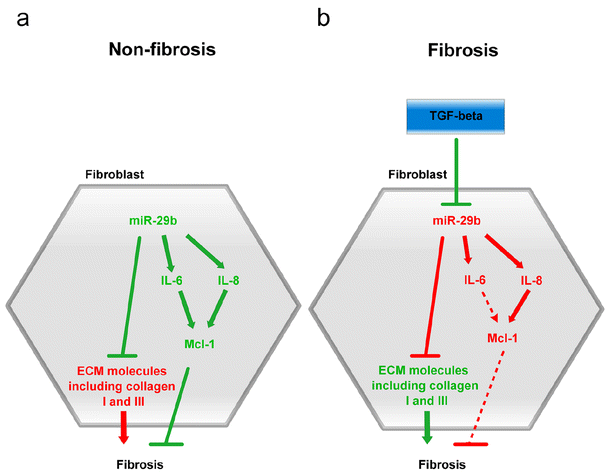

Supplement: Supplementary file 3 — (GIF 51 kb) [file 441_2017_2576_Fig9_ESM.gif]

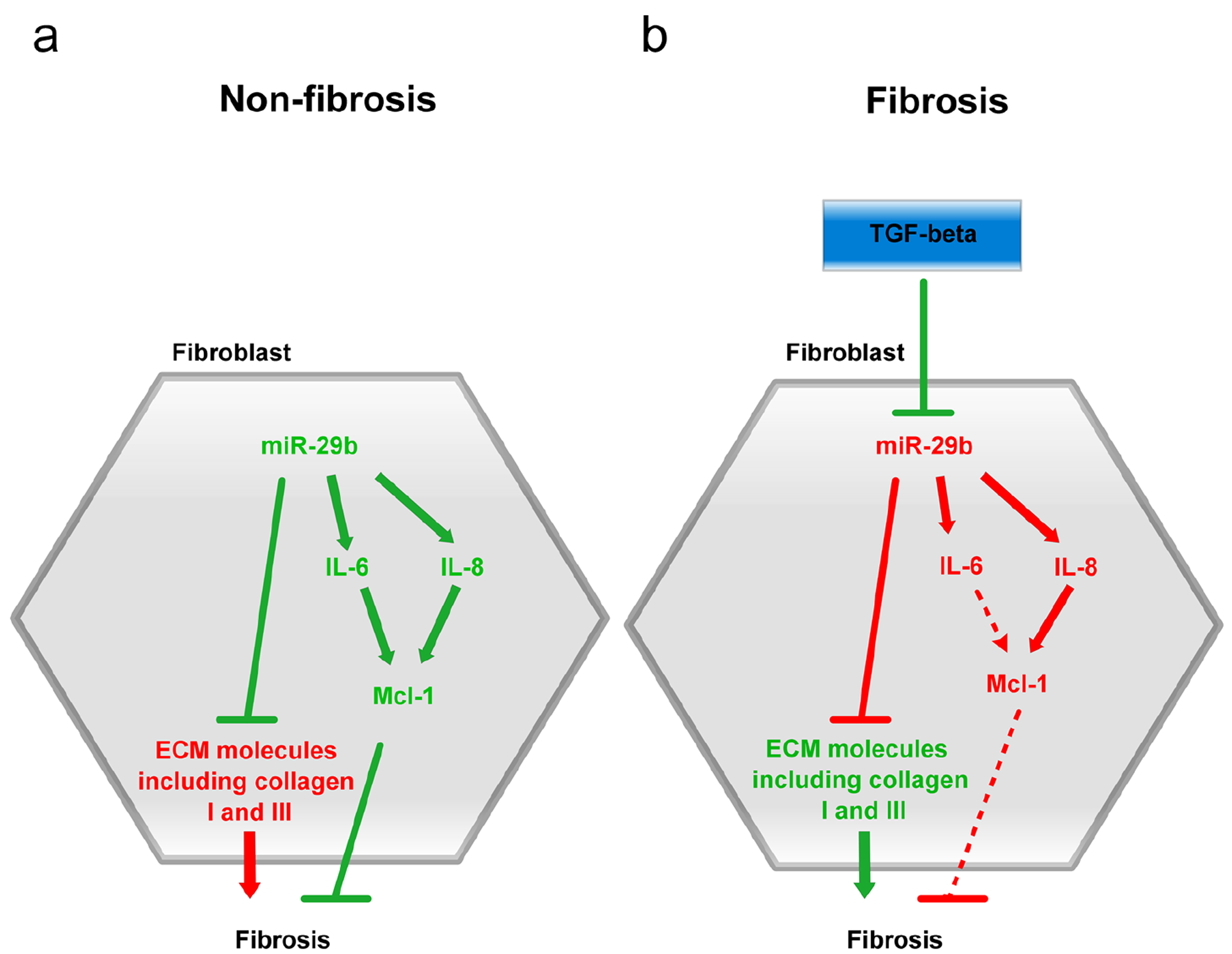

Supplement: Supplementary file 4 — High resolution image (TIF 9377 kb) [file 441_2017_2576_MOESM2_ESM.tif]
