# Supplementary material for: MCL-1 is modulated in Crohn’s disease fibrosis by miR-29b via IL-6 and IL-8
Source: Cell Tissue Res. 2017 Feb 11;368(2):325–35. doi: 10.1007/s00441-017-2576-1 (PMC5397660; doi:10.1007/s00441-017-2576-1)
Supplement: Supplementary file 5 — (DOC 28 kb) [file 441_2017_2576_MOESM3_ESM.doc]

**Supplementary Table 1. Densitometry of MCL-1 western blot. Densitometry of MCL-1S and MCL-1L positive bands was performed. Fold change of the MCL1L isoform is expressed relative to the MCL1-S isoform.**

| **Sample** | **Fold change** |
| --- | --- |
| HCT116 | 28.7 |
| DLD-1 | 2.0 |
| HT55 | 9.9 |
| NT29 | 7.9 |
| SW837 | 4.5 |
| VACO4S | 1.9 |
| CD fibroblasts | 3.7 |
